# Comment on Restrepo-Botero et al. Sum of Skinfold-Corrected Girths Correlates with Resting Energy Expenditure: Development of the NRG_CO_ Equation. *Nutrients* 2024, *16*, 3121

**DOI:** 10.3390/nu17111810

**Published:** 2025-05-27

**Authors:** Frank Carrera-Gil, Mikel Izquierdo, Robinson Ramírez-Vélez

**Affiliations:** 1 Departamento de Alimentación y Nutrición, Facultad de Ciencias de la Salud, Pontificia Universidad Javeriana Seccional Cali, Cali 760021, Colombia; 2 Navarrabiomed, Hospital Universitario de Navarra (HUN), Navarra Institute for Health Research (IdiSNA), Universidad Pública de Navarra (UPNA), 31006 Pamplona, Spain; mikel.izquierdo@gmail.com (M.I.); robin640@hotmail.com (R.R.-V.); 3 CIBER of Frailty and Healthy Aging (CIBERFES), Instituto de Salud Carlos III, 28029 Madrid, Spain

We read with interest the article published by Restrepo-Botero et al. [1], which aimed to evaluate the performance of three predictive equations (PEs) commonly used in clinical practice for estimating resting energy expenditure (REE): Harris–Benedict, Mifflin–St. Jeor, and FAO/WHO/UNU. Additionally, the authors developed a new equation to estimate REE in Colombian adults with moderate-to-high levels of physical activity. To assess the predictive accuracy of these equations, the authors compared the estimated REE with that measured using indirect calorimetry (IC). They employed correlation tests to analyze whether the estimated and measured REE values varied similarly and concordance tests to determine the quantitative match between the estimates and actual measurements, including an analysis of potential biases.

The study included 86 apparently healthy adults under 60 years of age, residing in two major cities and their metropolitan areas. Half of the participants were men, with a mean age of 27.5 ± 7.7 years and a BMI of 23.8 ± 3.65. The Harris–Benedict, Mifflin–St. Jeor, and FAO/WHO/UNU equations showed moderate correlations with REE but exhibited significant biases. Consequently, the authors developed a new predictive equation for REE by dividing participants into a development group (*n* = 71) and a validation group (*n* = 15). The new equation, which included body mass, sum of skinfolds, corrected thigh and calf perimeters, and age, yielded a coefficient of determination (R^2^) of 0.57. We appreciate the authors’ efforts, as this research enriches the limited body of evidence on the performance of PEs in the Colombian population. Additionally, we recognize the study’s contributions and emphasize key points for further exploration.

First, although standard tests were applied to determine the correlation and concordance between the estimated and measured REE values, the authors did not calculate the proportion of REE estimates differing by more than 10% from the measured value. This is a clinically relevant parameter that provides insight into the accuracy of REE predictors [2]. A difference exceeding 10% can lead to underfeeding or overfeeding [3,4], both of which are associated with adverse health outcomes [5].

Second, in PE validation studies, it is essential to account for the participants’ physiological state during IC. The authors reported that they followed the methodological criteria proposed in by the “Evidence Analysis Working Group” in 2006 [6], which includes ensuring that participants avoided thermogenic supplements, sleep or appetite suppressants, or other substances affecting REE during the 24 h preceding IC. However, it remains unclear whether the participants adhered to a standardized fasting period before REE measurement, or if there were any guidelines to avoid physical training during the 48 h prior to measurement.

Additionally, the method for REE measurement was not thoroughly described. For instance, there was no mention of urinary nitrogen excretion testing, which allows for nitrogen correction in protein oxidation calculations, leading to more accurate REE estimation in IC studies. Moreover, complementary data such as the duration of REE measurement and reports on variables like carbon dioxide production (VCO_2_), oxygen consumption (VO_2_), or respiratory quotient (RQ) were omitted. These variables are important for determining which macronutrients (carbohydrates, fats, or proteins) predominated as energy sources during IC. Without this information, it becomes challenging to interpret the energy expenditure data accurately, as physiological states such as recent food intake or exercise could influence the use of carbohydrates or fats, thus affecting the precision of the PE. Given that VO_2_ and VCO_2_ kinetics can be influenced by factors such as age and sex [7], it would be beneficial to describe the gas-exchange data selection method, which could provide more accurate REE measurements. These considerations are essential to assess the study’s internal validity.

Third, the newly developed equation presents limitations in terms of applicability and the reliability of its estimates. It relies on measurements of circumferences and skinfolds, which can be labor-intensive in clinical practice and prone to evaluator errors, thereby compromising its reproducibility and precision. Furthermore, the new equation showed a coefficient of determination of 0.57, which is lower than that of Mifflin–St. Jeor (R^2^ = 0.71) [8], suggesting that the proportion of inaccurate estimates (a difference greater than 10% from the measured REE) could be high. In a study of 433 Chilean individuals, we observed that the same PE evaluated in this study produced inaccurate estimates ranging from 36% to 70% [9]. Despite having a higher R^2^ than the new equation, the Mifflin–St. Jeor equation, which is based on anthropometric data less prone to evaluator errors, still produced inaccurate estimates in a significant proportion (36%) of Chilean participants [9]. Given that the study by Restrepo-Botero et al. [1] had a relatively small sample size, less precise and accurate results could be expected.

Finally, despite numerous PEs having been developed over more than a century, this ongoing effort has not yielded significant improvements in REE estimation due to the complexity of energy metabolism, which is difficult to capture with equations based solely on sociodemographic and anthropometric data. Therefore, while IC is not yet accessible in all settings, we recommend careful consideration of the methodological aspects discussed and advise using PEs, including the new one, with caution. We eagerly await further research that builds on these insights to identify other parameters that may enhance REE estimation.
